# Engineered Expression of Hepatocyte Growth Factor Activator Inhibitor-1 (HAI-1) Reduces the Growth of Bladder Cancer Cells

**DOI:** 10.3390/biomedicines13040871

**Published:** 2025-04-03

**Authors:** Yuichi Katayama, Takahiro Akioka, Shoichi Kimura, Masato Fujii, Takahiro Nagai, Takumi Kiwaki, Makiko Kawaguchi, Tsuyoshi Fukushima, Yuichiro Sato, Shoichiro Mukai, Toshiyuki Kamoto, Atsuro Sawada

**Affiliations:** 1 Department of Urology, Faculty of Medicine, University of Miyazaki, Miyazaki 889-1602, Japan; yuuichi_katayama@med.miyazaki-u.ac.jp (Y.K.);; 2 Section of Oncopathology and Morphological Pathology, Department of Pathology, Faculty of Medicine, University of Miyazaki, Miyazaki 889-1602, Japan

**Keywords:** bladder cancer, HAI-1, HAI-2, HGF, MET

## Abstract

**Background:** The function of hepatocyte growth factor activator inhibitor (HAI)-1 and HAI-2 in bladder cancer has not been well evaluated. In a previous study, we reported upregulated MET phosphorylation and decreased expression of HAI-1 in bladder cancer as poor prognostic factors. In this study, we analyzed the therapeutic effect of HAI-1 and HAI-2 on bladder cancer cells through the inhibition of MET phosphorylation. **Methods:** We established stable HAI-1 and HAI-2 overexpression KU-1 cell lines (HAI-1 OE and HAI-2 OE) and HAIs knockdown T24 cell lines (HAI-1 KD and HAI-2 KD). These cell lines were used for cell proliferation, migration, and invasion assay. Next, the cell lines were injected with human fibroblasts subcutaneously in mice, and inhibition of growth was evaluated. **Result:** Significant inhibition in cancer cell proliferation, motility, and invasiveness was observed in HAI-1 OE and HAI-2 OE compared with the mock in the presence of HGF zymogen, whereas significant upregulation in cancer cell proliferation, motility, and invasiveness was observed in HAI-1 KD and HAI-2 KD cells. In vivo analysis showed significant inhibition of cancer cell growth in HAI-1 OE. Although a tendency toward the inhibition of growth was observed in HAI-2 OE, statistical significance was not achieved. Phosphorylation of MET in cancer tissues was downregulated in both cell lines. **Conclusions:** HAI-1 may have the therapeutic potential to reduce the growth of bladder cancer through the inhibition of MET phosphorylation.

## 1. Introduction

Approximately 500,000 patients have been newly diagnosed with bladder cancer worldwide in recent years, and the number is increasing [1]. Patients with muscle-invasive blader cancer (MIBC) are treated by radical cystectomy and urinary diversion. MIBC remains lethal and the cancer-specific survival at five years for patients with pT2 and pT3 has been reported as 65–78% and 31–55%, respectively, because MIBC has the potential for metastasis [2]. MIBC predominantly metastasizes to lymph nodes, bone, lung, and liver, and metastasis is associated with poor prognosis [3]. Therefore, the development of more effective agents is essential. Recently, significant therapeutic efficacy of a new regimen of combination therapy with immune checkpoint inhibitor and enfortumab vedotin, a human nectin-4 antibody conjugated to the highly potent microtubule-disrupting agent, monomethyl auristatin, was reported [4,5].

Hepatocyte growth factor (HGF) is a multifunctional growth factor and is reported to have an important role in the progression of various cancers through the activation of MET, which is a specific receptor of HGF [6]. HGF is secreted as a nonfunctional zymogen (pro-HGF) from fibroblasts and activated proteolytically by matriptase, hepsin, and HGF activator [7]. Enzymatic activity is tightly regulated by HGF activator inhibitor type-1 (HAI-1) and HAI-2 in physiological condition; however, downregulation of HAIs and upregulation of HGF-activating proteases have been reported in various cancers [8,9]. The imbalance causes overactivation of HGF and leads to increased cancer cell proliferation, motility, and invasive activity through increased MET phosphorylation [10,11]. In cancer tissue, upregulation of the HGF/MET signaling axis occurs mainly in a paracrine fashion [12].

In a previous study, we demonstrated by immunohistochemical analysis that increased phosphorylation of MET and expression of matriptase, which is a specific activator of pro-HGF in MIBC, was significantly associated with poor prognosis [13]. In addition, downregulation of HAI-1 and upregulation of matriptase were also correlated with poor prognosis. Of interest, phosphorylation of MET and expression of HAI-1 were reciprocally observed in the majority of cancer tissues. In the HAI-1 downregulated area, upregulation of matripatese was also observed. It was hypothesed that downregulation of HAI-1 induced overactivation of pro-HGF by matriptase, which caused phosphorylation of MET in the pericancerous microenvironment of MIBC. This suggested that ligand-dependent activation of MET was critical in bladder cancer, and the inhibition of HGF activation by HAI-1 might have the potential to regulate the progression of cancer.

Although expression of HAI-2 in bladder cancer has been reported, the biological function and correlation with clinical features, including prognosis, have not been determined [14]. In the database of The Human Protein Atlas (http://www.proteinatlas.org/), bladder cancer patients with decreased HAI-2 expression tended to have a worse prognosis. In another urological cancer, increased expression of hepsin and decreased expression of HAI-2 have been reported to correlate with poor prognosis in patients with renal cell carcinoma (RCC) [15]. Promoter methylation of *SPINT2* gene was observed in 30–40% of RCC patients, and this is known to cause downregulation of HAI-2 [16]. In a previous study, we successfully confirmed inhibition of RCC cell growth by engineered expression of HAI-2 through both in vitro and in vivo analysis [17]. Therefore, we hypothesize that HAI-2 may also inhibit the growth of bladder cancer by the inhibition of pro-HGF activation.

As mentioned above, we hypothesized that inhibition of HGF activation by HAI-1 and HAI-2 may have potential in cancer control. In this study, we evaluated the therapeutic effect for bladder cancer cells by the inhibition of HGF activation through overexpression of HAIs.

## 2. Materials and Methods

### 2.1. Antibodies and Reagents

Antibodies specific to phospho-MET (Y1234/1235), MET, Matriptase, and alpha tubulin were purchased from Cell Signaling Technology (Danvers, MA, USA). Anti-human HGF antibody and HAI-1 ware purchased from Abcam (Cambridge, UK). Anti-human HAI-2 mouse mAb 2A6121 was prepared in our laboratory.

Recombinant human pro-HGF was purchased from R & D systems (Minneapolis, MN, USA). Recombinant human HGF was purchased from PeproTech (Cranbury, NJ, USA). Antibody against c-MET (Y1235 phosphorylated) purchased from Immuno-Biological Laboratories (Gunma, Japan) was used for immunohistochemistry.

### 2.2. Cells and Cell Culture

Human bladder cancer cell line KU-1 was kindly provided by Professor M. Oya (Keio University, Tokyo, Japan), and T24 was purchased from the American Type Culture Collection (Manassas, VA, USA). All these cells were cultured under typical conditions in RPMI 1640 (Invitrogen, Carlsbad, CA, USA), supplemented with 10% fetal bovine serum (FBS, Gibco BRL, Grand Island, NY, USA), at 37 degrees Celsius in a humidified atmosphere of 5% CO_2_.

### 2.3. RNA Extraction

Total cellular RNA was extracted from cells using an RNA Mini kit (Ambion, Paisley, OR, USA). Tissue was homogenized by bead crusher (µT-1) before extracting RNA. Total RNA was extracted using a PureLink RNA Mini Kit (Thermo Fisher SCIENTIFIC, Waltham, MA, USA) according to the manufacturer’s directions. Genes of interest were amplified from 2 mg DNase I-treated total RNAs using Thunderbird Reverse Transcriptase (Toyobo, Tokyo, Japan) and random primers.

### 2.4. Real-Time Quantitative PCR

Real-time RT-PCR analyses were performed with a Thermal Cycler Dice Real Time System II (Takara, Shiga, Japan). Reaction mixture (25 µL) containing 2 µL of cDNA template, 1 µL each of sense and anti-sense primers and 1 × SYBR Premix Ex Taq II (Takara, Shiga, Japan) were amplified as follows: held at 95 °C for 30 s and 40 cycles at 95 °C for 5 s, 60 °C for 30 s, and final dissociation stage (95 °C for 15 s, 60 °C for 30 s and 95 °C for 15 s). GAPDH was used as an internal control. The results were evaluated using the Thermal Cycler Dice Real Time System software program version 5.11 (Takara Bio, Shiga, Japan). The delta-delta Ct (ddCt) algorithm was used to analyze the relative changes in gene expression. Experiments were repeated in triplicate; GAPDH was used as an internal control. The primers were as follows: GAPDH forward, 5′-GCACCGTCAAGGCTGAGAAC-3′ and reverse, 5′-TGGTGAAGACGCCAGTGGA-3′; HGF forward, 5′-GTTCAATGTGGGACAAGAACATGG-3′ and reverse, 5′-GGATTTCGGCAGTAATTCTCATTCA-3′; HAI-1 forward, 5′-GGTGACACGGATGTCAGGGTA-3′ and reverse, 5′CACTGTCAGCTGGAACAGGTAGG3′; HAI-2 forward, 5′-GACGGAAACAGCAATAATTACCTGA-3′ and reverse, 5′-TTGAACATATCGCTGGAGTGGTC-3′; HGF forward, 5′-GTTCAATGTGGGACAAGAACATGG-3′ and reverse, 5′-GGATTTCGGCAGTAATTCTCATTCA-3′.

### 2.5. Protein Extraction and Immunoblot Analysis

Cells were washed twice with ice-cold PBS followed by incubation with the RIPA lysis buffer (Thermo Fisher Scientific, Waltham, MA, USA). The degenerated cells were scraped and collected into microcentrifuge tubes and centrifuged at 14,000 rpm at 4 °C for 3 min. The extracted protein was analyzed by immunoblot. The reaction samples were mixed with sodium dodecyl sulfate-polyacrylamide gel electrophoresis (SDS-PAGE) sample buffer and heated for 15 min at 75 °C. SDS-PAGE was performed under reducing conditions using 4–12% gradient gels. After electrophoresis, the sample proteins were transferred electrophoretically to Immobilon membranes (Millipore, Billerica, MA, USA). After blocking the nonspecific binding site with PDVF Blocking Reagent (TOYOBO), the membranes were incubated with primary antibody in buffer containing 1% bovine serum albumin (BSA) overnight at 4 °C, followed by four washes with the buffer and incubation with peroxidase-conjugated secondary antibody diluted in the buffer with 1% BSA for 1 h at room temperature. The labeled proteins were visualized with chemiluminescence reagent (PerkinElmer Life Sciences, Boston, MA, USA).

### 2.6. Establishment of Stably HAIs-Expressed KU-1 Cell Line, HAI-1 OE and HAI-2 OE

Human HAI-1 and HAI-2 cDNA was subcloned into pLenti6.3/TO/V5 (Life Technologies, Tokyo, Japan). Generation of the lentiviral particles and transfection into KU-1 cells were performed according to the manufacturer’s instructions. Then blasticidin-resistant stable HAI-1 and HAI-2 overexpression cell (HAI-1 OE, HAI-2 OE) pool and its control (KU-1pLentiMock: mock) were obtained.

### 2.7. Knockdown of SPINT1 and SPINT2 in T24 Cell Line

For transient silencing of the SPINT1 and SPINT2 gene, Stealth RNAi™ siRNA (ThermoFisher Scientific, Tokyo, Japan) was used. The sequences for SPINT1 were 5′-CCGCUUUACCUAUGGUGGUUGUUAC-3′ (siHAI-1) and -5′-GGGCUGUGACGGAAACAGCAAUAAU-3′ (si-HAI-2), whereas Stealth siRNA Negative Control (Thermo Fisher Scientific) was transfected as a control. Transfection of siRNA was performed using the lipofectamine RNAiMAX reagent (ThermoFisher). Then, HAI-1 and HAI-2 knockdown T24 cell (HAI-1 KD, HAI-2 KD) and its control (siNC) were made.

### 2.8. Cell Proliferation Assay

HAI-1 OE, HAI-2 OE, and the mock cells were cultured for 24 h in FBS-free medium prior to assay. The cells were seeded in a 96-well plate at a density of 1 × 10^4^ cells/100 μL in FBS-free medium. After 24 h incubation, pro-HGF (50 ng/mL) was added. After the addition of CellTiter 96 Aqueous One Solution (20 µL, Promega, Madison, WI, USA), the absorbance of each well at 490 nm was measured [3-(4,5-dimethylthiazol-2-yl)-5-(3-carboxymethoxyphenyl)-2-(4-sulfophenyl)-2H-tetrazolium proliferation assay: MTS-assay]. Cell proliferation was measured at 0 h, 24 h, 48 h, and 72 h. All the experiments were performed in triplicate, and the results were expressed as the mean of three separate experiments. The same procedures were also performed for HAI-1 KD, HAI-2 KD, and siNC.

### 2.9. Wound Healing Assay

HAI-1 OE, HAI-2 OE, and the mock cells were cultured for 24 h prior to assay. The cells were seeded in 6-well plates and cultured until they reached confluence. A wound was then created by manually scraping the cell monolayer with a 200 μL pipette tip. The cultures were washed twice with PBS to remove floating cells. The cells were then incubated in FBS-free medium and pro-HGF (50 ng/mL) was added. The wound area was measured at start and 0, 24, and 48 h. The percent closure was determined as follows: percent closure (%) = migrated cell surface area/total surface area × 100. The closure rate of each cell line was compared using the paired t-test. The same procedures were also performed for HAI-1 KD, HAI-2 KD, and siNC.

### 2.10. Cell Invasion Assay

Invasion assay was performed following the protocol for 24-well Corning BioCoat Matrigel Invasion Chamber (Corning, MA, USA). HAI-1 OE, HAI-2 OE, and the mock cells were maintained in medium with 0.1% bovine serum albumin (BSA, Roche, Penzberg, Germany) for use in the invasion assay. Each cell line was cultured with BSA 48 h prior to assay. Cells were harvested and re-suspended in medium with 0.1% BSA at 2 × 10^6^ cells/mL, and then 1 × 10^6^ cells/500 µL were seeded in each well. After 48-h incubation with recombinant pro-HGF (final concentration in culture 50 ng/mL), the invaded cells were counted and compared using the paired t-test. The same procedures were also performed for HAI-1 KD, HAI-2 KD, and siNC.

### 2.11. Animal Experiments

All experiments involving laboratory animals were performed in accordance with the Guidelines for Experiments of Miyazaki University (produced by Animal Experimentation Committee of Miyazaki University, approval code: 2022-510-2, approval date: 1 April 2023). Female athymic nude (BALB/AJcl-nu/nu) mice (Kyudo, Tosu-shi, Japan) were maintained under germ-free conditions until 5 weeks old. For subcutaneous injection, HAI-1 OE, HAI-2 OE, and mock cells were cultured, and then detached by trypsin EDTA at 80–90% confluence before resuspension in PBS to a final concentration of 5 × 10^6^/100 µL. The mice were anesthetized with isoflurane inhalation, and 5 × 10^6^ of cancer cells with 5 × 10^6^ of MRC5/100 µL PBS of these cells were injected right subcutaneously into mice. Tumor volumes were measured with a caliper using the formula a × b2 × 0.52, where a is the largest diameter and b is the smallest diameter perpendicular to a tumor.

### 2.12. Immunohistochemistry

Formalin-fixed paraffin-embedded sections were prepared according to the routine method. For immunohistochemistry, sections were processed for antigen retrieval (microwave in 10 mM citrate buffer, pH 6.0 for 10 min), followed by treatment with 3% H_2_O_2_ in methanol for 10 min and washed in tris-buffered saline (TBS) twice. After blocking in 3% bovine serum albumin and 5% goat serum in phosphate-buffered saline for 2 h at room temperature, the sections were incubated with primary antibodies overnight at 4 °C. Negative controls did not include the primary antibody. Sections were then washed in TBS and incubated with Envison-labeled polymer reagent (DAKO, Santa Clara, CA, USA) for 30 min at room temperature. Sections were exposed to nickel, cobalt-3, 3-diaminobenzidine (Immunopure Metal Enhanced DAB Substrate Kit; Piece, Rockford, IL, USA), and counterstained with hematoxylin.

### 2.13. Statistical Analysis

SPSS statics, version 25.0 (SPSS, II, Chicago, IL, USA), was used to assess the statistical parameters. Differences between each group were compared by the paired t-test. The statistical tests were two-sided and *p*-values < 0.05 were considered statistically significant.

## 3. Results

*3.1. mRNA Expression of HGF/MET-Related Molecules in Bladder Cancer Cell Lines, T-24 and KU-1*

**Figure 1:**
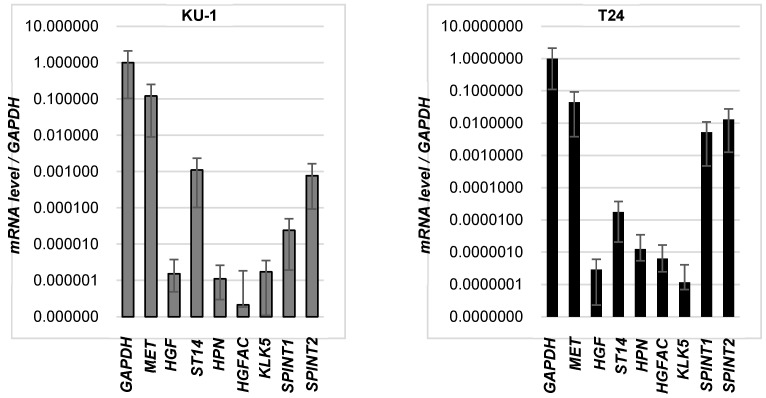
mRNA expression of HGF-related molecules in bladder cancer cell lines. Expression of human *HGF*, *MET*, *ST14* (encoding matriptase), *HPN* (encoding hepsin), *HGFAC* (encoding HGF activator), *KLK5* (encoding kallikrein-related peptidase 5), *SPINT1* (encoding HAI-1) and *SPINT2* (encoding HAI-2) in KU-1 and T-24 was determined by real-time RT-qPCR.

Expression of HGF/MET signaling pathway-related molecules in T-24 and KU-1 was determined by real-time RT-PCR (Figure 1). *HGF*, *MET,* and *ST-14* encoding HGF-activating protease, matriptase, were upregulated in KU-1 compared with T-24. And *KLK5* encoding kallikrein-related peptidase 5 (KLK5), which is an activator of HGFA zymogen, was upregulated in KU-1. SPINT-1 and SPINT-2 encoding HAI-1, 2, which are specific inhibitors of HGF-activating proteases and KLK5 (inhibited by HAI-1), were downregulated in KU-1 compared with T-24.

## 3.2. Inhibition of Pro-HGF Activation by HAIs

**Figure 2:**
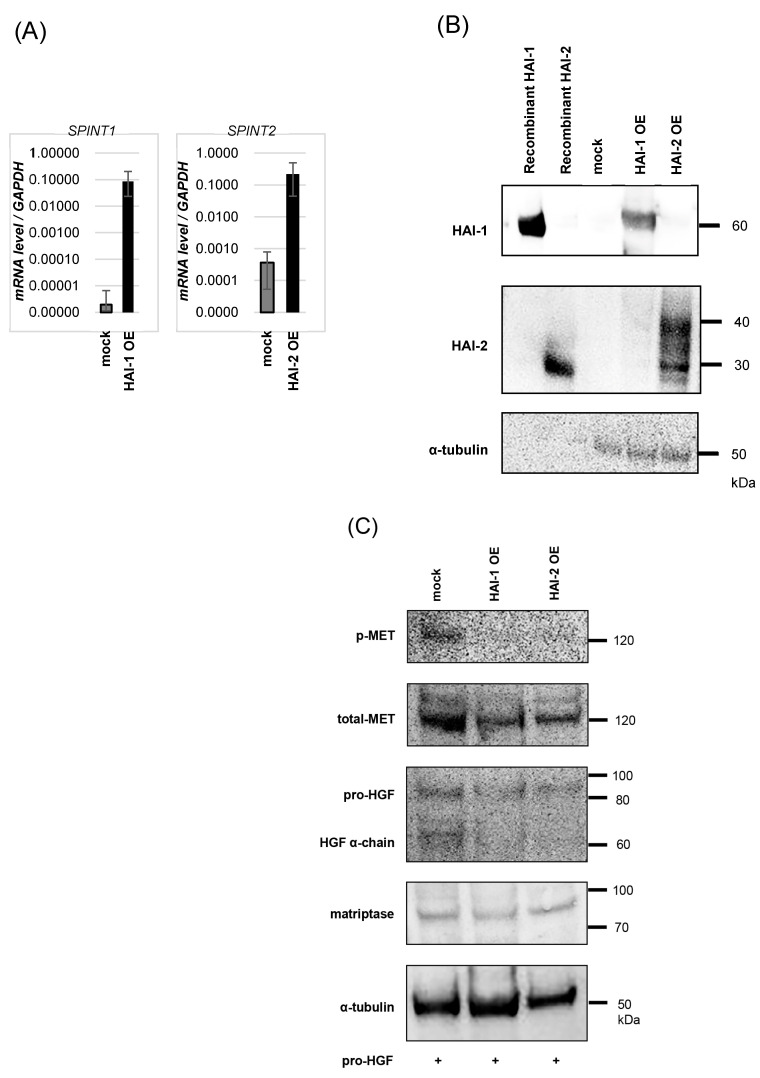
Establishment of HAI-1 and HAI-2 overexpressing KU-1 cell lines, and inhibition of HGF activation by HAIs. (**A**) Expression of *SPINT1* and *SPINT2* in HAI-1 OE and HAI-2 OE was confirmed by real-time RT-qPCR. (**B**) Expression of HAIs protein was determined by immunoblots. (**C**) Phosphorylation of MET and expression of total-MET, HGF, and α-tubulin were determined by immunoblot analysis. HAI-1 OE and HAI-2 OE cells were pre-cultured in FBS-free medium for 24 h., and then human recombinant pro-HGF (50 ng/mL) was added. After incubation at 37 °C for 2 h, the proteins were extracted.

According to the previous results, we established that HAI-1 and HAI-2 overexpressed KU-1 cell lines, HAI-1 OE, and HAI-2 OE. Expression of HAI-1 and HAI-2 was confirmed by real-time RT-qPCR and immunoblots (Figure 2A,B). Next, the inhibitory effect on pro-HGF activation was determined. As shown in Figure, the inhibition of HGF activation and significant downregulation of MET phosphorylation were observed in both HAI-1 OE and HAI-2 OE (Figure 2C).

## 3.3. Inhibition of Bladder Cancer Cell Proliferation, Migration, and Invasion by HAIs

**Figure 3:**
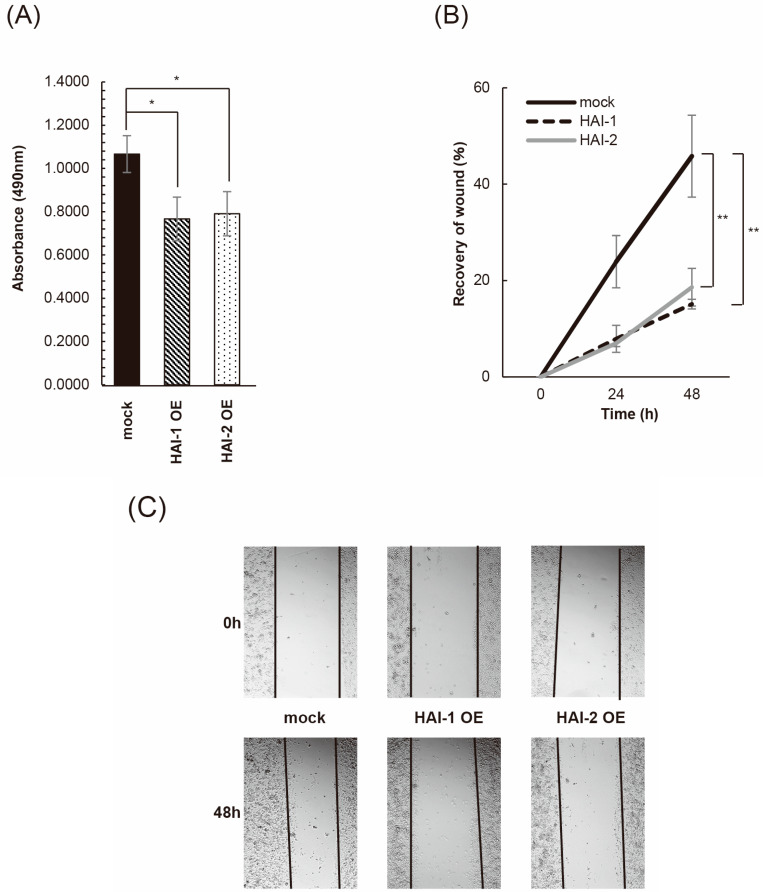

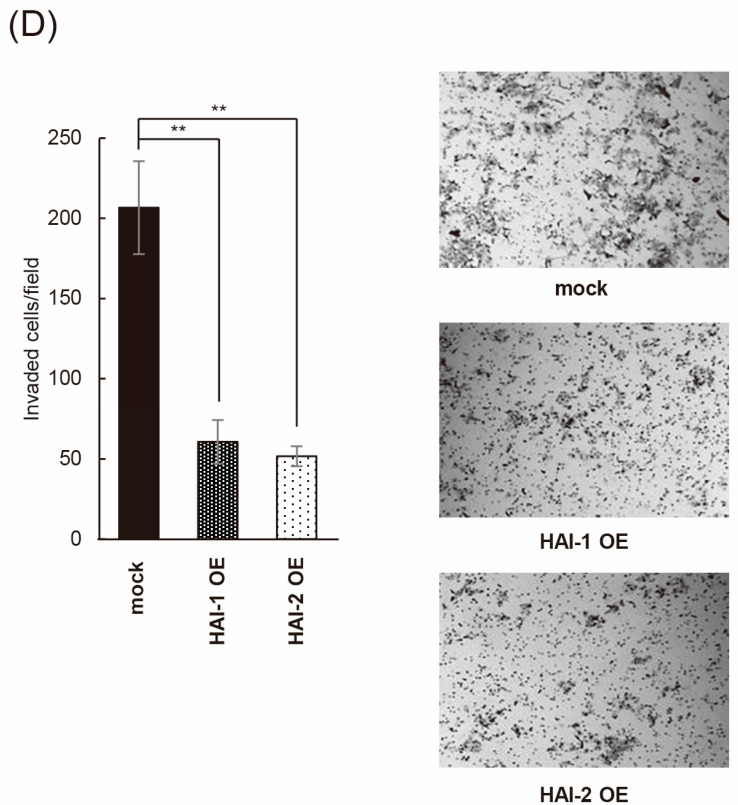
Inhibition of KU-1 cell proliferation, migration, and invasion by HAIs. (**A**) Result of MTS-assay is shown. Cell proliferation was measured at 0 h, 24 h, 48 h, and 72 h. All experiments were performed in triplicate. * *p* < 0.05. (**B**) Result of wound healing assay is shown. Extent of cell movement was calculated at the indicated periods, and displayed as mean ± SD of three independent experiments. The wound area was measured at start and 0, 24, and 48 h. The percent closure was determined as follows: percent closure (%) = migrated cell surface area/total surface area × 100. The closure rate of each cell line was compared using the paired t-test. ** *p* < 0.01. (**C**) Photomicrographs of HAI-1 OE and HAI-2 OE cells in wound healing assay are shown. (**D**) Result of invasion assay (**left**: invaded cells/field, **right**: representative photomicrograph of invaded cells) is shown. Values are mean number ± SD of invaded cells per high-power field in triplicate experiments. After 48-h incubation with pro-HGF (final concentration in culture 40 ng/mL), invaded cells were counted and compared using the paired *t*-test. ** *p* < 0.01.

The effect on cell proliferation was analyzed by MTS-assay. HAI-1 OE and HAI-2 OE cells were significantly decreased in cell proliferation in the presence of pro-HGF (Figure 3A). The wound healing assay also revealed a significant decrease in cell motility in both HAI-1 OE and HAI-2 OE in the presence of pro-HGF (Figure 3B,C). Next, we performed an invasion assay in the presence of pro-HGF. As a result, the invasive activity of KU-1 cell was largely downregulated by the expression of both HAI1 and HAI-2 (Figure 3D). Statistical significance was also confirmed.

## 3.4. Effect on Bladder Cancer Cell Proliferation, Migration, and Invasion by Knockdown of HAIs

**Figure 4:**
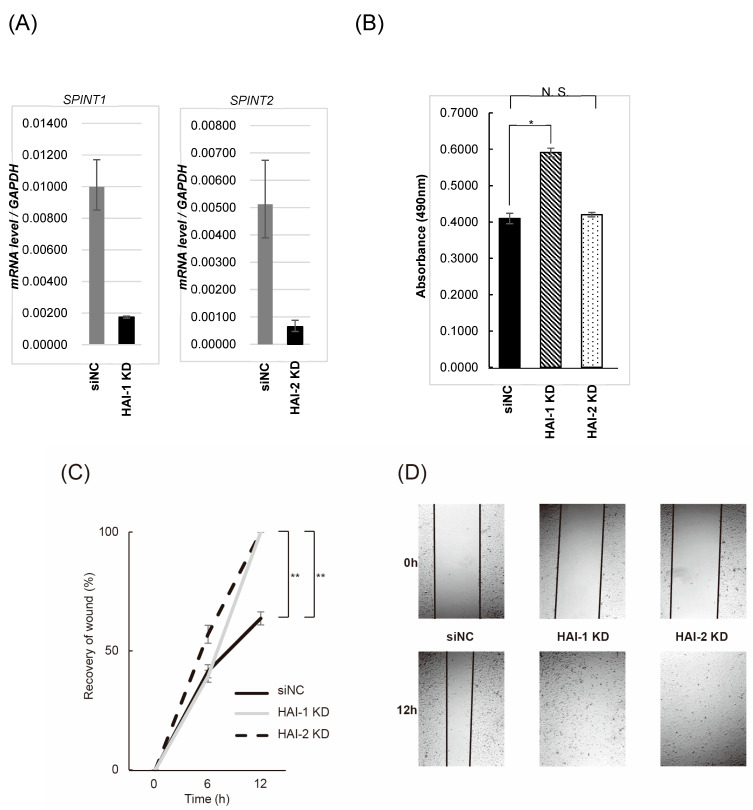

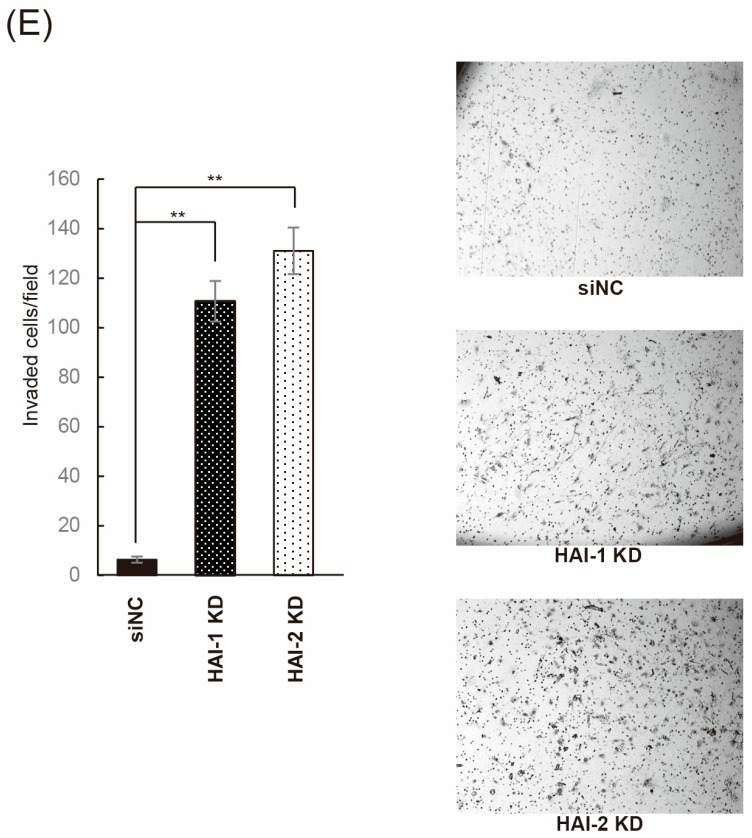
Effect for T24 cell proliferation, migration and invasion by knockdown of HAIs. (**A**) Expression of SPINT1 and SPINT 2 in HAI-1 KD and HAI-2 KD was confirmed by RT-qPCR. (**B**) Result of MTS-assay is shown. Cell proliferation was measured at 0 h, 24 h, 48 h, and 72 h. All experiments were performed in triplicate. * *p* < 0.05. (**C**) Result of wound healing assay is shown. Extent of cell movement was calculated at the indicated periods, and displayed as mean ± SD of three independent experiments. The wound area was measured at start and 0, 6, and 12 h. The percent closure was determined as follows: percent closure (%) = migrated cell surface area/total surface area × 100. The closure rate of each cell line was compared using the paired t-test. ** *p* < 0.01. (**D**) Photomicrographs of HAI-1 KD and HAI-2 KD cells in wound healing assay are shown. (**E**) Result of invasion assay (**left**: invaded cells/field, **right**: representative photomicrograph of invaded cells) is shown. Values are mean number ± SD of invaded cells per high-power field in triplicate experiments. After 48-h incubation with pro-HGF (final concentration in culture 40 ng/mL), invaded cells were counted and compared using the paired *t*-test. ** *p* < 0.01.

Initially, we established HAI-1 knockdown T24 cell (HAI-1 KD) and HAI-2 knockdown T24 cell (HAI-2 KD). Decreased expression of HAIs mRNA was confirmed by RT-qPCR (Figure 4A). The effect on cell proliferation was analyzed by MTS-assay. HAI-1 KD was significantly increased in cell proliferation in the presence of pro-HGF; however, significance was not observed in HAI-2 KD compared with control (Figure 4B). Wound healing assay revealed significant increase in cell motility in both HAI-1 KD and HAI-2 KD in the presence of pro-HGF (Figure 4C,D). Next, we performed invasion assay in the presence of pro-HGF. As a result, invasive activity of T24 cell was upregulated by knockdown of both HAI-1 and HAI-2 (Figure 4E). Statistical significance was also confirmed.

## 3.5. Therapeutic Effect of HAIs on Growth of Bladder Cancer, In Vivo Analysis

**Figure 5:**
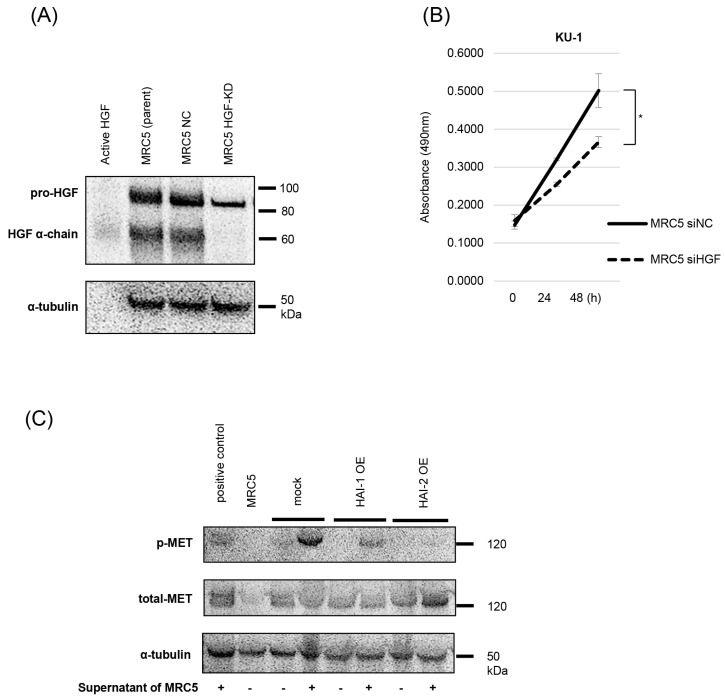
Inhibition of human fibroblast (MRC5)-derived HGF activation by HAIs. (**A**) Expression of HGF protein is shown by immunoblot. Expression of HGF in MRC5 (parent and siNC) and the downregulation in HGF-knock down MRC5 (MRC5 HGF-KD) are confirmed. Recombinat active HGF was used for positive control. (**B**) Result of cell proliferation assay (MTS-assay) is shown. Supernatant of MRC5 siNC and MRC5 siHGF are added to KU-1 cells, and proliferation is determined. * *p* < 0.05. (**C**) Phosphorylation of MET by MRC5-derived HGF is analyzed. Supernatant of MRC5 induced phosphorylation of MET is observed in mock, whereas the phosphorylation is downregulated in HAI-1 OE and HAI-2 OE. PC3 cells were used for positive control.

Next, we undertook an in vivo analysis through the subcutaneous implantation of KU-1 cells in mouse model. Because of insufficient activity of mouse HGF to human MET, co-implantation of human fibroblasts MRC5, which involves a high expression of pro-HGF, was planned. After confirmation of HGF expression in MRC5, we also prepared HGF-knock down MRC5 (Figure 5A). The effect of MRC5-produced HGF (supernatant) was also determined. Compared with control (MRC5 siNC), supernatant of HGF-knock down MRC5 significantly downregulated cell proliferation of KU-1 (Figure 5B). In addition, supernatant of MRC5 upregulated phosphorylation of MET (mock), and the effect was inhibited by the expression of HAI-1 and HAI-2 (HAI-1 OE and HAI-2 OE), suggesting inhibition of MRC5-produced pro-HGF activation by HAIs (Figure 5C).

**Figure 6:**
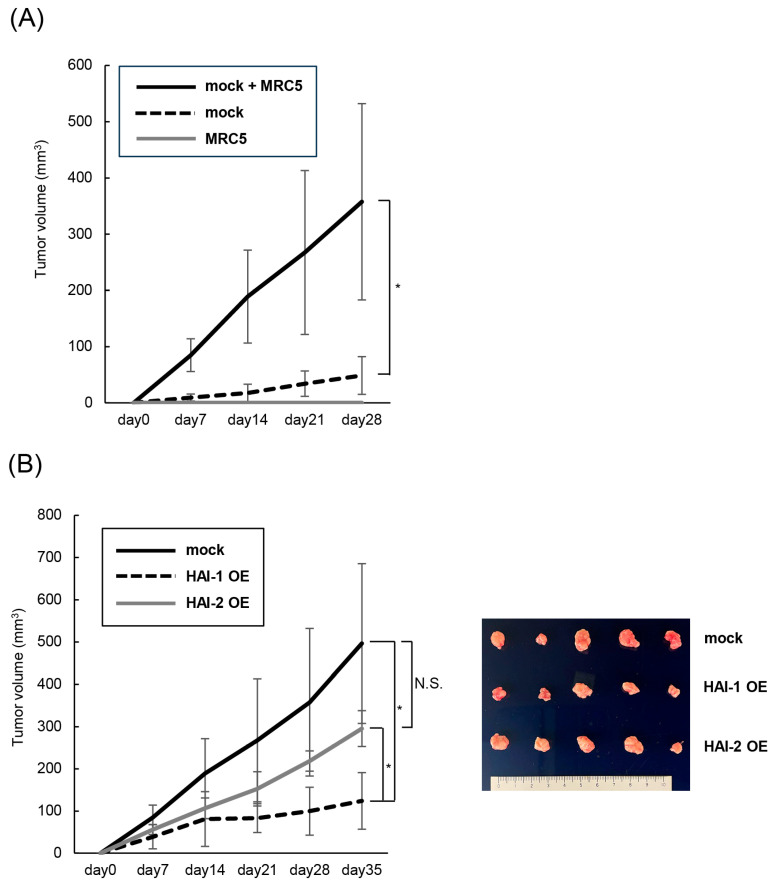

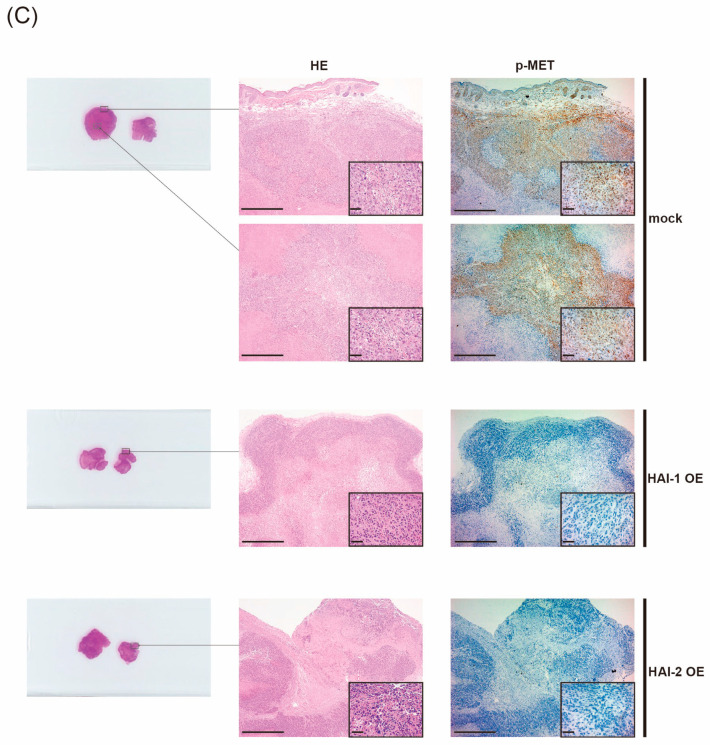
Inhibition of fibroblast-derived HGF activation by HAIs: co-implantation with MRC5 in mouse. (**A**) Growth of KU-1 cells implanted subcutaneously with or without MRC5 is shown. Significant growth of KU-1 cells is observed by co-implantation with MRC5. * *p* < 0.05. (n = 5, each group) (**B**) Growth of HAI-1 OE, HAI-2 OE and mock with MRC5 is shown. Significant downregulation of cancer cell growth is observed in HAI-1 OE cells compared with mock and HAI-2 OE cells. * *p* < 0.05. (n = 5, each group) No statistical significance is observed in HAI-2 OE cells compared with mock. NS: not significant. Gross appearance of tumor tissues is shown (**right**). Horizonal lines show upper to lower, mock, HAI-1 OE, HAI-2 OE. (**C**) Representative histological findings are shown. Upper two horizonal lines are mock, middle line is HAI-1 OE, and lower line is HAI-2 OE. Vertical columns are from left to right, loupe, hematoxylin-eosin (HE) staining and immunohistochemical staining of phosphorylation of MET (*p*-MET). Appearance of high magnification is shown in inset. Scale bar = 500 μm, 50 μm (inset).

Of interest, co-implantation of KU-1 and MRC5 in the subcutaneous tissue of mice revealed significant upregulation of cancer cell growth compared with single implantation of KU-1 (Figure 6A). We then determined the inhibitory effect of HAIs in this model. Three types of KU-1 cells, including mock, HAI-1 OE, and HAI-2 OE, were co-implanted with MRC5 subcutaneously. As a result, cancer cell growth was significantly downregulated in HAI-1 OE compared with mock and HAI-2 OE (Figure 6B). Cancer cell growth tended to downregulate in HAI-2 OE compared with mock; however, statistical significance was not observed.

Histologically, we confirmed that all the specimens were composed of viable cancer cells with necrotic area in part. No apparent difference in the extent of the necrotic area was observed. Phosphorylation of MET was upregulated in mock, but significantly downregulated in HAI-1 OE and HAI-2 OE immunohistochemically (Figure 6C).

## 4. Discussion

HAIs are known as single-pass transmembrane Kunitz-type serine protease inhibitors of pro-HGF-activating proteases [18]. HAI-1 is encoded by *SPINT1* gene, which is located on chromosome 15q15.1 [19]. A significant inhibitory effect for HGFA, hepsin, matriptase, and KLK5 has been reported due to the specific inhibitor domain binding [11]. Downregulation of HAI-1 is reported to be correlated with poor prognosis in patients with prostate, breast, ovarian, and endometrial cancer [11,20,21,22,23,24]. In addition, both upregulation of matriptase and downregulation of HAI-1 in patients with bladder cancer are reported to have poor prognosis [13]. In this study, overexpression of HAI-1 inhibited phosphorylation of MET in KU-1 cells and reduced the proliferation, motility, and invasive growth in the presence of pro-HGF, and knockdown of HAI-1 upregulated the proliferation, motility, and invasive growth of T24 cells. In addition, subcutaneous co-implantation with MRC5 showed a significant therapeutic effect for the growth of KU-1 cells in the mouse model. HAI-1 also has the potential to inhibit KLK5, which is an activator of HGFA [25]. Increased expression of KLK5 in invasive bladder cancer has been reported and correlated with invasiveness. KU-1 expressed KLK5, and knock down of KLK5 inhibited invasion [26]. Therefore, overexpression of HAI-1 might play an important role in the inhibition of pro-HGF-activating proteases and KLK5, which induced inhibition of cancer cell growth. In the data of the Cancer Genome Atlas in Gene Expression Profiling Interactive Analysis (GEPIA, http://gepia.cancer-pku.cn), high expression of KLK5 also correlated with poor prognosis (Supplementary Figure S1); however, no correlation was observed in HAI-1 and HAI-2. In addition, no apparent statistical correlation was also observed between the expression of *HGF, MET, ST14, HPN,* and *HGFAC* and the prognosis of patients with bladder cancer by GEPIA (Supplementary Figure S1).

HAI-2 is encoded by the *SPINT2* gene, which is located on chromosome 19q 13.2 [8,27]. *SPINT2* gene promoter has been reported to be hypermethylated in various cancers, including hepatocellular carcinoma (HCC), RCC, melanoma, gastric cancer, and esophageal squamous cell carcinoma [8]. HAI-2 is also downregulated in prostate cancer; however, no apparent methylation of *SPINT2* promoter has been reported [28]. In the analysis of prostate cancer, it has been reported that downregulation of HAI-2 induced progression of prostate cancer by upregulation of matriptase; however, engineered expression of HAI-2 reduced growth [29]. An inhibitory effect on cancer progression by re-expression of HAI2 has been reported in various cancers, including HCC, medulloblastoma, glioblastoma, breast cancer, melanoma, cervical cancer, ovarian cancer, and RCC [14]. In this study, *SPINT2* was downregulated in KU-1 cells, and engineered expression of HAI-2 reduced phosphorylation of MET through reduced activation of pro-HGF. In addition, overexpressed HAI-2 reduced the proliferation, migration, and invasion of KU-1 cells, and knockdown of HAI-2 also upregulated the proliferation, motility, and invasive growth of T24 cells. In vivo analysis showed a tendency of a similar inhibitory effect on the growth of KU-1 cells; however, statistical significance was not observed. Unfortunately, we could not explain a potential reason for this result in this study. Further investigation, including an accurate comparison of the inhibitory potential (in vitro and in vivo) and a spectrum of target proteases between HAI-1 and HAI-2 may be necessary. Although expression of HAI-2 in bladder cancer has been reported, functional analysis in bladder cancer has not been reported. To the best to our knowledge, this study is the first to discuss HAI-2-induced inhibition of bladder cancer cell growth.

Recently, the significant therapeutic effect of SRI31215, a novel small molecule inhibitor of pro-HGF activation (inhibitor of hepsin, matriptase and HGF activator: similar function with HAIs), has been reported in MET-amplified non-small cell lung and gastric cancer cells [30,31,32]. In addition, it was also reported that the pharmacological effect of MET inhibitors on the growth of cancer cells was attenuated under HGF-rich condition, and additional inhibition of inhibitor of HGF-activating proteases overcame the resistance [30]. The results suggested the significance of HGF-targeted therapy in cancer treatment and overcoming the resistance of MET inhibitor. Therefore, the development of such synthetic inhibitors with a similar inhibitory function to HAIs is recommended, either as single or combined use with an MET inhibitor.

In our in vivo analysis, we evaluated the significance of human fibroblasts (MRC5), which expressed human pro-HGF. As a result, the growth of KU-1 cells was significantly upregulated in co-implantation with MRC5 compared with KU-1 implantation without MRC5 (Figure 5A). Insufficient biological activity of mouse HGF to human MET has been reported [30]. Our result is consistent with the report, and this procedure may be useful for the accurate evaluation of HGF/MET signaling axis in vivo study.

This study has some limitations. First, an expression analysis of HAIs with a large number of clinical samples will be necessary to clarify the clinical significance of HAIs in patients with bladder cancer. Second, it is still unclear why the difference in effect on cancer cells occurs between HAI-1 and HAI-2 in part of the in vitro and in vivo analyses. Further investigation, including an accurate comparison of the inhibitory potential for proteases and a search for other target molecules of HAIs, may be necessary. Third, the in vivo analysis results showed a slight variation in tumor size. Although the significance was observed between HAI-1 OE and control, further examination with more mouse models will be necessary to arrive at an accurate conclusion (the preliminary data are shown in Supplementary Figure S2).

## 5. Conclusions

We established that HAI-1 and HAI-2 overexpressed bladder cancer cell line HAI-1 OE and HAI-2 OE. In the presence of pro-HGF, phosphorylation of MET was downregulated, and the proliferation, motility, and invasive growth of both cell lines were also significantly downregulated in both cell lines. Finally, the therapeutic effect of HAI-1 was observed by subcutaneous co-implantation in mice with human fibroblast cell line, MRC5. HAI-1 may have the potential to reduce bladder cancer cell growth.

## Data Availability

The original contributions presented in this study are included in the article and in the Supplementary Materials. Further inquiries can be directed to the corresponding author.

## Supplementary Materials

The following supporting information can be downloaded at: https://www.mdpi.com/article/10.3390/biomedicines13040871/s1, Figure S1. Kaplan-Meier curves for survival of HAIs-related molecules from GEPIA (Bladder Cancer). Figure S2. Growth of HAI-1 OE, HAI-2 OE and mock with MRC5 is shown.

## Abbreviations

The following abbreviations are used in this manuscript:

HAI-1	Hepatocyte growth factor inhibitor type 1
HAI-2	Hepatocyte growth factor inhibitor type 2
MET	Hepatocyte growth factor receptor
HGF	Hepatocyte growth factor
Pro-HGF	HGF zymogen
MIBC	Muscle-invasive bladder cancer
SPINT	HAI coding gene
KLK5	Human kallikrein-related peptidase 5
HPN	Hepsin coding gene
ST-14	Matriptase coding gene
