# Supplementary material for: Engineered Expression of Hepatocyte Growth Factor Activator Inhibitor-1 (HAI-1) Reduces the Growth of Bladder Cancer Cells
Source: Biomedicines. 2025 Apr 3;13(4):871. doi: 10.3390/biomedicines13040871 (PMC12024841; doi:10.3390/biomedicines13040871)
Supplement: Supplementary file 1 [file biomedicines-13-00871-s001.zip › biomedicines-3525308-supplementary.pdf]

Supplemental Figure 1

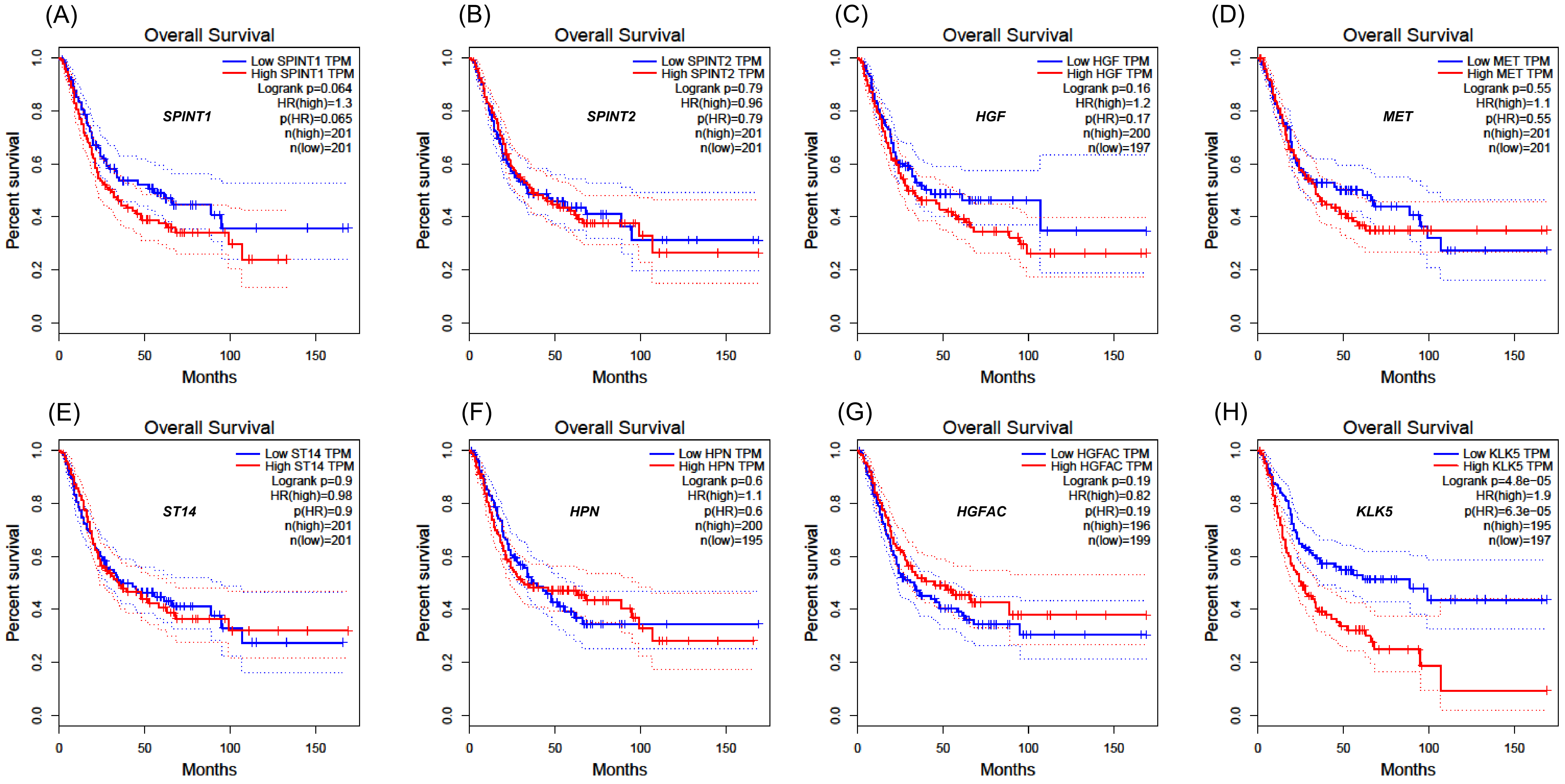

Supplemental Figure S1

Kaplan-Meier curves for survival of HAIs-related molecules from GEPIA (Bladder Cancer). (A) Survival analysis for *SPINT1*. (B) Survival analysis for *SPINT2.* (C) Survival analysis for *HGF.* (D) Survival analysis for *MET.* (E) Survival analysis for *ST14.* (F) Survival analysis for *HPN.* (G) Survival analysis for *HGFAC.* (H) Survival analysis for *KLK5.*

Supplemental Figure 2

(A) (B)

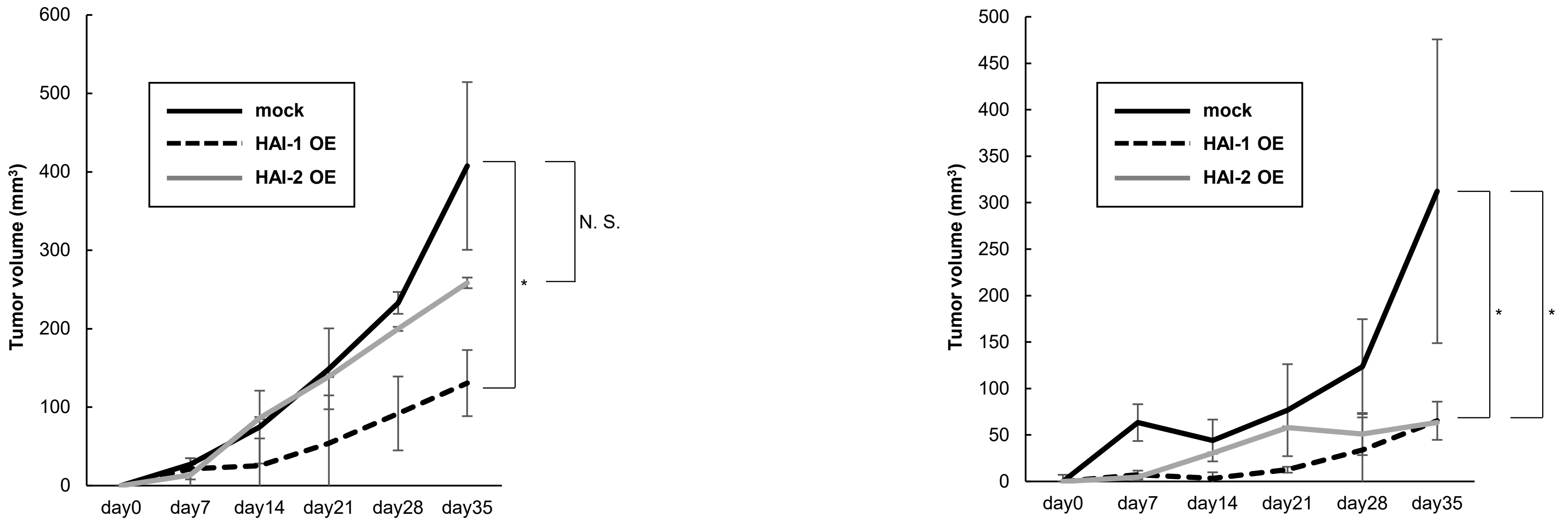

Supplemental Figure S2

Growth of HAI-1 OE, HAI-2 OE and mock with MRC5 is shown.
(A) 2.5×10^6 of cancer cells with 5×10^6 of MRC5/100 μl PBS of these cells were injected right subcutaneously into mice (n=2, each group).
(B) 5×10^6 of cancer cells with 5×10^6 of MRC5/100 μl PBS of these cells were injected right subcutaneously into mice (n=2, each group).
Tumor volumes were measured with a caliper using the formula, a×b2×0.52, where a is the largest diameter and b is the smallest diameter perpendicular to a tumor. Significance was observed between HAI-1 OE and mock (A). Significance was observed in both HAI-1 OE versus mock and HAI-2 OE versus mock (B). *$P<0.05$.
